# Body composition and ankle-brachial index in Ghanaians with asymptomatic peripheral arterial disease in a tertiary hospital

**DOI:** 10.1186/s40608-016-0107-3

**Published:** 2016-05-13

**Authors:** Kwame Yeboah, Peter Puplampu, Ernest Yorke, Daniel A. Antwi, Ben Gyan, Albert G. B. Amoah

**Affiliations:** Department of Physiology, School of Biomedical & Allied Health Sciences, University of Ghana, P.O. Box KB 143, Accra, Ghana; Department of Medicine & Therapeutics, School of Medicine & Dentistry, University of Ghana, Accra, Ghana; National Diabetes Management & Research Centre, Korle-Bu Teaching Hospital, Accra, Ghana; Department of Immunology, Noguchi Memorial Institute of Medical Research, University of Ghana, Accra, Ghana

**Keywords:** Obesity, Ankle brachial index, Peripheral arterial disease, Diabetes, Ghana

## Abstract

**Background:**

Ankle-brachial index (ABI) and indices of obesity are both use to indicate cardiovascular risk. However, association between body composition indices and ABI, a measure of peripheral arterial disease, is inconsistent in various study reports. In this study, we investigated the relationship between ABI and general and central indices of obesity in Ghanaians without history of cardiovascular diseases.

**Method:**

In a case–control design, ABI was measured in a total of 623 subjects and categorised into PAD (ABI ≤ 0.9, *n* = 261) and non-PAD (ABI > 0.9, *n* = 362) groups. Anthropometric indices, BMI, waist circumference (WC), waist-hip ratio (WHR) and waist-height ratio (WHtR) were also measured.

**Results:**

PAD subjects had higher mean BMI (29.8 ± 8.7 vs. 26.5 ± 7.6 kg/m^2^, *p* = 0.043) and waist circumference (95 ± 15 vs. 92 ± 24 cm, *p* = 0.034) than non-PAD subjects. In multivariable logistic regression models, having BMI ≥ 30 kg/m^2^ increased the odds of both unilateral [OR (95 % CI): 2 (1.14–3.51), *p* < 0.01] and overall PAD [2 (1.22–3.27), *p* < 0.01].

**Conclusion:**

In indigenous Ghanaians in our study, PAD participants had higher BMI and waist circumference than non-PAD participants. Also, halving BMI ≥ 30 kg/m^2^ was associated with twofold increase in the odds of PAD.

**Electronic supplementary material:**

The online version of this article (doi:10.1186/s40608-016-0107-3) contains supplementary material, which is available to authorized users.

## Background

Peripheral arterial disease (PAD) is one of the debilitating cardiovascular diseases (CVDs) among diabetes patients and the older general population [1]. Most PAD cases remain undetected until later stages of the disease when clinical symptoms become apparent [2]. Ankle-brachial index (ABI), the ratio of systolic blood pressures of the arteries of the ankle to the arteries of the upper arm, is a simple, objective and non-invasive method of screening for PAD [3]. The major risk factors for PAD are diabetes and smoking [2], however, the prevalence of smoking in Ghana is relatively low [4]. The prevalence of symptomatic and asymptomatic PAD in diabetes patients far outweighs that of non-diabetic individuals [5]. Obesity, characterized by excess body fat, is recognized as important factor for development of CVDs [6] including PAD [7]. Obesity is assessed as body mass index (BMI) for generalized adiposity, and waist circumference (WC), waist-hip ratio (WHR) and waist-height ratio (WHtR) for central adiposity [8]. Body composition and adiposity exhibit ethnic diversity, with indigenous Africans having different morphology compared to Caucasians [9]. Also, there is no agreement on the best index of adiposity associated with CVDs. For instance, as some studies reported that, compared to BMI, indices of central obesity such as WC, WHR and WHtR are better determinant of CVD morbidity and mortality [10, 11]; other investigators found no superiority of these measures of abdominal obesity to BMI in CVD risk prediction [12]. In this study, we investigated the association between various indices of obesity and PAD in subjects without any history of CVD. We hypothesize that obesity increases the odds of PAD in our study population.

## Methods

This study was case control design, conducted within the period of August, 2009 to June, 2010, at the Korle-Bu Teaching Hospital in Accra, which is a 1500-bed tertiary hospital and serves as the main referral hospital in Ghana. ABI was measured in all the study population, which were selected from two sources: (1) diabetes patients, selected systematically as every 3rd consecutive patient visiting the diabetes clinic and consented to take part in the study, and (2) non-diabetic individuals, invited from the surrounding communities and conveniently recruited into the study. All the study participants were categorised as PAD and non-PAD based on the ABI values. Individuals with stiff incompressible arteries (ABI > 1.3), symptoms of classical intermittent claudication, history/medication of CVDs and those unable to comprehend and comply with the protocol requirements (psychological and/or cognitive disorders, failure to cooperate, failure to sign the informed consent document) were excluded from the study. The sample size was estimated to detect correlation coefficient of 0.2 and 0.3 between BMI and ABI in PAD and non-PAD subjects respectively; minimum of 186 subjects were required to power the study at 80 % with type I error rate of 5 %. In all, 261 PAD patients and 362 non-PAD subjects satisfied the inclusion criteria and were recruited into the study. The study was approved by the University of Ghana Medical School Ethical and Protocol Review Committee (Protocol ID number: MS-Et/M.2 – P.2.10/2009-2010) and all participants gave written informed consent after the procedures involved in the study were thoroughly explained to them, following the general recommendations of the Declaration of Helsinki.

Weight, height, waist and hip circumferences were measured using standard protocol [8]. Briefly, body weight was determined on twice using a homologated electronic scale (Seca 770) following due calibration (precision ± 0.1 kg), with the patient wearing light clothing with shoes removed. Height was also measured with a portable system (Seca 222) with the patient shoeless in the upright position. Body mass index (BMI) was calculated as weight (kg) divided by height squared (m^2^). BMI < 18.5 Kg/m^2^ were considered underweight, those within the range of 25–29.9 Kg/m^2^ were considered overweight and BMI ≥ 30 Kg/m^2^ were considered obese. Waist circumference was measured with non-elastic tape measure at the upper border of the iliac crest, parallel to the floor without compressing the skin. The reading was taken at the end of a normal breath, and WHR < 0.9 & WC < 102 cm for males and WHR <0.85 & WC < 88 cm for females were considered normal. WHtR was calculated as height divided by WC, with WHtR <0.5 considered normal [8].

Blood pressure was measured three times, with a validated Blood Pressure Monitor (Omron 991X, Omron Health Care, Japan), at the right upper arm of participants with an appropriate cuff size, after at least 5 min rest, seated comfortably with arm and back support. Hypertension was defined as subjects with BP ≥ 140/90 mmHg and/or on antihypertensive medication.

Ankle and brachial blood pressures were measured in all participants after a minimum of 5 min rest in a supine position on an examination table in a temperature controlled room. Blood pressure cuffs were applied to bare ankles with the midpoint of the bladder over the ankle arteries (dorsalis pedis and posterior tibial arteries) approximately 2 cm above the medial malleolus. Systolic blood pressure in both arms and ankles were obtained using an 8-MHz handheld Doppler (LifeDop® 250, Summit Doppler). For each limb, the cuff was inflated rapidly to the maximal inflation level and deflated at a rate of 2 mmHg per second until the systolic blood pressure became audible. Measurements were obtained in the following order: right arm, right ankle, left ankle, and left arm. ABI was calculated for each leg as the ratio of the higher of the systolic blood pressure in the ankle (dorsalis pedis and posterior tibia arteries) divided by the higher of systolic blood pressure in the arm. ABI >0.9 was considered normal and PAD was defined as ABI ≤ 0.9 in at least one leg [3]. Since none of the participants had history/medication of CVDs, the level of atherosclerotic burden in PAD participants was estimated as unilateral (ABI ≤ 0.9 in one leg) or bilateral (ABI ≤ 0.9 in both legs) [13, 14]. Bilateral PAD has been reported to indicate more diffuse vaso-occlusion, as well as reduced arterial compliance, compared to unilateral PAD [13].

The data was analysed using IBM SPSS Version 20. Socio-demographic and clinical characteristics of study participants were analysed with student’s *t*-test for continuous variables and *χ*^2^ test for categorical variables. Association between anthropometric indices and leg-specific ABI were assessed using multivariable regression with covariates forced into the model. The relationship between obesity and PAD status was analysed using logistic regression model with adjustment of covariates. *p* < 0.05 was considered statistically significant.

## Results

**Fig. 1:**
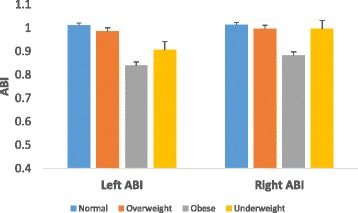
Mean ABI across various BMI groups

**Fig. 2:**
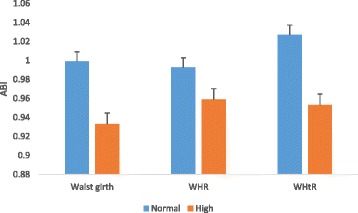
Mean ABI of the right leg across various central adiposity categorisation

**Fig. 3:**
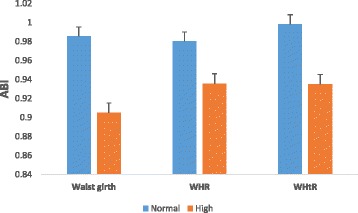
Mean ABI of the left leg across various central adiposity categorisation

Of the 261 participants screened to have low ABI (≤0.9), 79 (12.6 %) participants had in the right leg alone, 96 (15.4 %) in the left leg only and 86 (13.8 %) in both legs. Compared to participants without PAD, PAD participants had higher BMI, waist circumference, systolic BP and heart rate, and also, most of them were on insulin (Table 1). Comparison of clinical characteristics between diabetes patients and non-diabetic participants in the study population is shown in Additional file 1: Table S1. Across the various BMI groups, mean ABIs were significantly lower in obese (BMI ≥30 kg/m^2^) participants in both right and left legs (Fig. 1). Similarly, with respect to the measures of central adiposity, participants with high levels of waist circumference, WHR and WHtR had lower mean ABIs in both right (Fig. 2) and left (Fig. 3) leg values. Comparison of adjusted mean values of various anthropometric characteristics between patients with unilateral, bilateral and overall PAD is shown in Additional file 1: Table S2.

**Table 1:** Socio-demographic and clinical characteristics by PAD status

	All participants (*n* = 623)	PAD (*n* = 261)	Non-PAD (*n* = 362)	p
Age, yrs	54.1 ± 10.6	54.2 ± 10.7	53.5 ± 10.2	0.493
Female, n (%)	331 (51.3)	163 (26.2)	168 (27)	0.01
Diabetes, n (%)	358 (57.4)	192 (30.8)	166 (26.6)	0.245
Duration of diabetes, yrs	6.8 ± 5.9	7.4 ± 6.2	6.2 ± 5.2	0.068
Hypertension, n (%)	292 (46.9)	143 (23)	149 (23.9)	<0.001
BMI, kg/m^2^	28 ± 8.2	29.8 ± 8.7	26.5 ± 7.6	0.043
Height, cm	163 ± 11	163 ± 12	164 ± 12	0.402
Waist circumference, cm	93 ± 13	95 ± 15	92 ± 24	0.034
Hip circumference, cm	102 ± 12	103 ± 14	101 ± 10	0.131
Waist-hip ratio	0.92 ± 0.09	0.92 ± 0.09	0.92 ± 0.23	0.873
Waist-height ratio	0.56 ± 0.07	0.59 ± 0.09	0.54 ± 0.07	0.855
Systolic BP, mmHg	133 ± 27	135 ± 25	130 ± 26	0.042
Diastolic BP, mmHg	80 ± 13	83 ± 13	79 ± 13	0.25
Pulse BP, mmHg	55 ± 19	56 ± 21	54 ± 15	0.318
Mean BP, mmHg	98 ± 15	98 ± 16	98 ± 15	0.296
Heart rate, bpm	70 ± 13	79 ± 16	63 ± 15	0.008
Employed, n (%)				0.127
Unemployed	232 (37.2)	108 (17.2)	124 (20)	
Part-time employment	38 (6.1)	14 (2.2)	24 (3.9)	
Full employment	353 (56.7)	136 (21.8)	217 (34.9)	
Smoking, n (%)				0.22
Current	32 (5.1)	11 (1.7)	21 (3.4)	
Former	111 (17.9)	44 (7)	67 ((10.9)	
Never	480 (77)	203 (32.5)	277 (44.5)	
Alcohol intake, n (%)	189 (30.3)	118 (18.9)	71 (11.4)	0.237
Insulin use, n (%)	177 (28.4)	121 (19.4)	56 (9)	0.01

**Table 2:** Multivariable linear regression analysis of Anthropometric Indices and ABI

	Unadjusted model		Model 1		Model 2	
	β	p	β	p	β	p
All participants						
BMI	−0.16	<0.001	−0.14	0.001	−0.15	0.003
WC	−0.17	<0.001	−0.15	<0.001	−0.13	0.003
WHR	−0.07	0.108	−0.07	0.148	−0.03	0.434
WHtR	−0.18	<0.001	−0.17	<0.001	−0.15	0.002
Diabetes patients						
BMI	−0.15	<0.001	−0.14	<0.001	−0.2	0.01
WC	−0.15	0.006	−0.11	0.042	−0.09	0.119
WHR	0.02	0.684	0.04	0.503	0.05	0.344
WHtR	−0.15	0.005	−0.12	0.044	−0.132	0.075
Non-diabetic subjects						
BMI	−0.26	<0.001	−0.22	<0.001	−0.21	0.001
WC	−0.22	<0.001	−0.19	0.002	−0.19	0.003
WHR	−0.04	0.536	−0.06	0.315	−0.07	0.293
WHtR	−0.26	<0.001	−0.21	0.002	−0.2	0.003

Model 1: Adjusted for age and gender

Model 2: Adjusted for age, gender, height, mean BP, employment status, diabetes and hypertension status and insulin use

*BMI* body mass index, *WC* Waist circumference, *WHR* waist-hip ratio, *WHtR* waist-height ratio

In multiple regression analysis using the lower of either right or left leg ABI as dependent variable, BMI, waist circumference and waist-height ratio were associated with ABI in both crude and adjusted models in diabetes and non-diabetic participants, as well as overall participants (Table 2). In multivariable logistic regression analysis, male gender was associated with reduction in, whereas hypertension increased, the odds of overall and leg-specific PADs. Insulin usage increased the odds of overall PAD, and cigarette usage also, increased the odds of bilateral PAD. Generalized obesity (BMI ≥ 30 kg/m^2^) was associated with increased odds of unilateral and overall PADs and high waist circumference increased the odds of bilateral PAD (Table 3).

**Table 3:** Multivariable logistic regression of obesity and PAD status

	PAD (at least one leg)	Unilateral PAD	Bilateral PAD
	β (95 % CI)	β (95 % CI)	β (95 % CI)
Age	0.99 (0.98–1.01)	0.99 (0.98–1.01)	1.01 (0.98–1.04)
Height	1.36 (0.25–7.51)	1.41 (0.25–7.84)	0.248 (0.03–2.32)
Mean BP	0.99 (0.98–1)	0.99 (0.98–1.01)	0.99 (0.97–1.01)
Male gender	0.58 (0.38–0.88)	0.62 (0.42–0.92)	0.49 (0.26–0.93)
Employment (Ref: Full–time)			
Part-time	0.78 (0.38–1.62)	0.77 (0.37–1.59)	1.33 (0.75–4.87)
Unemployed	1.27 (0.86–1.86)	1.27 (0.86–1.86)	1.31 (0.5–3.43)
Diabetes	1.01 (0.65–1.55)	1.04 (0.68–1.59)	0.89 (0.46–1.72)
Insulin use	1.92 (1.03–3.72)	1.85 (0.96–3.57)	1.89 (0.46–1.72)
Hypertension	2.11 (1.29–3.45)	1.82 (1.03–3.19)	3.19 (1.56–6.52)
Alcohol intake	1.08 (0.74–1.58)	0.89 (0.57–1.39)	1.58 (0.91–2.73)
Cigarette usage	1.21 (0.76–1.95)	0.93 (0.93–3.86)	2.15 (1.09–4.25)
Anthropometry^a^			
BMI groups (Ref: Normal)			
Overweight	1.19 (0.78–1.82)	1.68 (0.72–1.88)	1.25 (0.64–2.42)
Obese	2 (1.22–3.27)	2 (1.14–3.51)	1.94 (0.94–3.99)
WC (Ref: Normal)			
High	1.28 (0.84–1.95)	1.39 (0.78–2.24)	1.34 (0.61–2.1)
WHR (Ref: Normal)			
High	1.08 (0.72–1.62)	1.12 (0.7–1.77)	0.99 (0.54–1.86)
WHtR (Ref: Normal)			
High	1.07 (0.68–1.67)	1.02 (0.63–1.67)	1.38 (0.64–2.98)

^a^Adjusted for age, gender, height, mean BP, diabetes status, hypertension status, diabetes and hypertension medication, smoking and alcohol intake

*WC* waist circumference, *WHR* waist-hip ratio; *WHtR* waist-height ratio

## Discussion

The main objective of this study was to investigate the association between indices of obesity and ABI-diagnosed PAD. Due to high burden of PAD in diabetes patients [15], we included substantial number of diabetes patients in order to obtain significant cases of PAD to compare with non-PAD controls. The findings of this study showed that, compared to non-PAD subjects, mean BMI and Waist circumference were higher in PAD subjects. Halving a higher BMI (≥30 kg/m^2^) doubled the likelihood of PAD after adjustment of some risk factors.

The relationship between BMI, an index of generalized adiposity, and PAD reported in various studies is inconsistent. Similar to our findings, other studies reported association between PAD (low ABI) and BMI. In Cardiovascular Health Study, Ix et al. reported that significant association between BMI and ABI exist only in non-smokers with good health status [16]. Also, the Hearts of Brazil studies reported significant association between ABI-diagnosed PAD versus BMI, waist circumference and waist-hip ratio [17]. However, in contrast to our findings, BMI ≥ 30 kg/m^2^ was reported to be protective against PAD in Spanish population [18]. Others studies found no association between ABI and BMI [19, 20]. This lack of association between BMI and PAD may be explained by the masking effect of the U-shaped relationship between ABI and cardiovascular risk factors; individuals with ABI < 0.9 and ABI > 1.3 have similar high CVD risk. Therefore, in the design of this study, exclusion of subjects with ABI > 1.3 may partially explain the significant association between ABI and anthropometric indices.

Variation exist in blacks and Caucasian populations with respect to the burden of obesity and PAD. Comparative analysis of black participants in the Jackson Heart Study and Caucasians in Framingham Heart Study showed that African-Americans have higher burden of obesity and CVD risk factors, yet, the influence of obesity to CVD burden is lesser than that of Caucasians [21]. Generally, blacks have high level of adiposity independent of diabetes and hypertension, which may also be associated with higher odds of PAD [22]. Moreover, ethnic comparison of ABI in African-Americans and non-Hispanic whites reported in both the GENOA [23] and MESA [24] studies showed that African-Americans had lower mean ABI in both studies, but similar prevalence of PAD after adjustment of confounders.

Indices of abdominal adiposity, waist circumference and WHR, have been reported to be associated with PAD in both prospective [11, 25] and cross-sectional studies [10, 26]. BMI, waist circumference, WHR and WHtR are indices use to estimate amount and distribution of body fat, which is linked to the pathophysiology of vascular dysfunction [27]. In obese subjects, adipose tissue is known to release several metabolites, cytokines and hormones that modulates liver-derived lipoproteins, clotting factors and inflammatory factors, which affect the atherogenic environment of the vessel wall, favouring the development of atherosclerotic vascular diseases such as PAD [28]. Adipose tissue–derived factors also influence gene expression and function of cells involved in vascular homeostasis, such as endothelial cells, arterial smooth muscle cells, and monocytes/macrophages [6].

The findings of our study show that means of WHR were similar between PAD and non-PAD subjects, and in contrast to other studies [27]. Also, higher waist circumference and WHR, biomarkers of central obesity, were not associated with PAD. Compared to Caucasians, body composition of black ethnicity is associated differently with CVD risk profile. With similar BMI and waist circumference, computed tomographic analysis showed that African-Americans had lower visceral fat than Hispanics and Caucasians [9]. WHR and waist circumference are indices of fat accumulation in the abdominal viscera and intra-abdominal organs, with or without reference to fat accumulation in gluteo-femoral region respectively. As a marker of visceral fat accumulation, WHR and waist circumference reflect the relative inability of subcutaneous adipose tissue to act as a protective metabolic sink for the clearance and storage of extra energy derived from dietary triglycerides, leading to ectopic fat deposition in visceral adipose depots [29]. Visceral adipose tissue and its adipose-tissue resident macrophages produce more pro-inflamatory cytokines like tumor necrosis factor-alpha and interleukin-6, and less athero-protective adiponectin, making visceral fat measurement a proxy for the level of atherogenicity [30]. However, subcutaneous and visceral fat distribution exhibit ethnic variation, with Caucasians accumulating more fat in the abdominal region which can be readily mobilised, as blacks, on the contrary, accumulate fat in the subcutaneous region. In addition, visceral fat accumulation correlates with waist circumference better in Caucasians than Africans [9, 29]. This may partially explain the non-significant association between indices of central adiposity and risk of PAD prevalence in our study population.

The strength of this study is the relatively large sample size of equivalent proportion of PAD and non-PAD subjects, selected by systematic random sampling, and ABI measured in temperature controlled room. Also, subjects with stiff arteries, intermittent claudication and history of CVDs were excluded from the study. The major limitations include the study being a cross sectional clinic-based design, and hence, we cannot infer causality. Also, plasma biomarkers were not analysed to study the pathophysiological mechanisms relating obesity to PAD.

## Conclusion

The findings of this study show that, in Ghanaian without any history of CVD, PAD patients had higher BMI and waist circumference than non-PAD subjects. In addition, halving a BMI ≥ 30 kg/m^2^ increased the odds of PAD by twofold.

## Ethics, consent and permissions

The study was conducted in accordance with the Declaration of Helsinki and ethical approval was granted by the University of Ghana Medical School Ethical and Protocol Review Committee (Protocol ID number: MS-Et/M.3 – P.2.10/2009-2010). All participants gave written informed consent after thorough explanation of the procedures involved.

## Availability of data and materials

The dataset supporting the conclusions of this article is readily available for systematic review & meta-analysis upon request.

## Additional file

Additional file 1: Table S1. Socio-demographic and clinical characteristics by diabetes status. **Table S2.** Adjusted means of anthropometric indices by leg-specific PAD status. (DOCX 15 kb)

## Abbreviations

ABI: ankle-brachial index
BMI: body mass index
CVDs: cardiovascular diseases
DM: diabetes mellitus
PAD: peripheral arterial disease
