# Supplementary material for: Body composition and ankle-brachial index in Ghanaians with asymptomatic peripheral arterial disease in a tertiary hospital
Source: BMC Obes. 2016 May 13;3:27. doi: 10.1186/s40608-016-0107-3 (PMC4866025; doi:10.1186/s40608-016-0107-3)
Supplement: Additional file 1: Table S1. — Socio-demographic and clinical characteristics by diabetes status. Table S2. Adjusted means of anthropometric indices by leg-specific PAD status. (DOCX 15 kb) [file 40608_2016_107_MOESM1_ESM.docx]

**Supplementary (Web only)**

Table S1 Socio-demographic and clinical characteristics by diabetes status.

|  | All Participants (n=623) | DM  (n=358) | Non-DM (n=265) | p |
| --- | --- | --- | --- | --- |
| **Age, yrs** | **54.2±10.5** | **55.7±10.3** | **52±10.3** | **<0.001** |
| Female n (%) | 331 (53.1) | 192 (30.9) | 138 (22.2) | 0.738 |
| **Hypertension, n (%)** | **292 (46.9)** | **255 (40.9)** | **37 (5.9)** | **<0.001** |
| **BMI, kg/m^2^** | **28**±8.2 | **28.8**±9 | **26.3**±**5.8** | **<0.001** |
| **Height, cm** | **163±11** | **163±16** | **164±8** | **0.048** |
| **Waist circumference, cm** | **93**±13 | **94**±12 | **91**±15 | **0.002** |
| Hip circumference, cm | 102±12 | 101±12 | 102±12 | 0.136 |
| **Waist-hip ratio** | **0.92±0.09** | **0.94±0.08** | **0.89±0.09** | **<0.001** |
| **Waist-height ratio** | **0.57±0.1** | **0.58±0.1** | **0.56±0.1** | **<0.001** |
| **Waist-height-hip ratio** | **0.56±0.07** | **0.58±0.08** | **0.54±0.06** | **<0.001** |
| **Systolic BP, mmHg** | **133**±27 | **143**±24 | **122**±**22** | **<0.001** |
| Diastolic BP, mmHg | **80±13** | **84±13** | **74±10** | **<0.001** |
| Pulse BP, mmHg | **55±19** | **59±17** | **48±19** | **<0.001** |
| **Mean BP, mmHg** | **98±15** | **104±15** | **90±11** | **<0.001** |
| **Heart rate, bpm** | **70±13** | **75±11** | **59±17** | **<0.001** |
| **ABI** | **0.97±0.16** | **0.92±0.18** | **1.02±0.13** | **<0.001** |
| **PAD (any leg)** | **261 (41.8)** | **190 (30.5)** | **71 (11.3)** | **0.038** |
| Unilateral | 175 (28) | 123 (19.7) | 52 (8.3) | 0.254 |
| **Bilateral** | **86 (13.8)** | **67 (10.8)** | **19 (3)** | **0.04** |
| Employed |  |  |  | 0.151 |
| Unemployed | 232 (37.2) | 150 (24.1) | 88 (14.1) |  |
| Part-time employment | 38 (6.1) | 21 (3.4) | 17 (2.5) |  |
| Full employment | 353 (56.7) | 211 (31.6) | 169 (25.3) |  |
| Smoking, n (%) |  |  |  | 0.179 |
| Current | 32 (5.1) | 9 (1.4) | 23 (3.7) |  |
| Former | 111 (17.9) | 81 (13) | 30 (4.9) |  |
| Never | 480 (77) | 268 (43) | 212 (34) |  |
| Second-hand smoking | 93 (15) | 59 (9.5) | 34 (5.5) | 0.282 |
| Alcohol intake, n (%) | 189 (30.3) | 96 (15.4) | 93 (14.9) | 0.12 |

Table S2 Adjusted means of anthropometric indices by leg-specific PAD status

|  | No PAD | PAD | | p |
| --- | --- | --- | --- | --- |
|  |  | Unilateral | Bilateral |  |
| BMI | 27.1±7 | 28.7±9.1 | 30.5±9.6^#^ | <0.001 |
| WC | 91±24 | 95±14^#^ | 98±16^*^ | <0.001 |
| WHR | 0.91±0.22 | 0.93±0.09 | 0.93±0.09 | 0.892 |
| WHtR | 0.56±0.07 | 0.59±0.08^#^ | 0.61±0.11^*^ | 0.001 |

BMI, body mass index; WC, Waist circumference; WHR waist-hip ratio; WHtR, waist-height ratio.

Adjusted for age, gender, height, mean BP, employment status, diabetes and hypertension status and insulin use.

#: p<0.05, compared to non-PAD participants

*: p<0.05, compared to both non-PAD and single leg PAD participants
